# Prevalence of myopia in Chinese children and adolescents: a systematic review and meta-analysis

**DOI:** 10.7189/jogh.16.04056

**Published:** 2026-03-20

**Authors:** Hui Gao, Jiaqi Ma, Zhirong Liu, Jiaqi Wang, Wenjun Wang, Lu Ye

**Affiliations:** 1 Xi’an People's Hospital (Xi’an Fourth Hospital), Xi’an, China; 2 College of Food Science and Engineering, Northwest A&F University, Yangling, China

## Abstract

**Background:**

There is currently a lack of nationally representative assessments of myopia based on gold-standard cycloplegic refraction among Chinese children and adolescents. Therefore, we aimed to systematically review and meta-analyse the prevalence of myopia, diagnosed by cycloplegic refraction (spherical equivalent ≤−0.50 D), among Chinese children and adolescents, and examine its distribution across sexes, age groups, time periods, and geographic regions.

**Methods:**

We searched PubMed, Web of Science, Science Direct, CNKI, and Wanfang for population- or school-based studies published between January 2020 and March 2025 that used cycloplegic refraction. We calculated the pooled prevalence and 95% confidence intervals using a meta-analysis, with subgroup analyses by sex, educational stage, time periods, and regions. We assessed publication bias using Egger's and Begg's tests.

**Results:**

The overall myopia prevalence among 34 studies with 139 765 participants was 30.1%. Prevalence was higher in females (28.6%) than males (26.0%) and increased markedly with education stage from 4.2% in kindergarten, 28.4% in primary school, 64.1% in junior high, and 81.0% in high school. Temporally, prevalence peaked in 2016 (57.5%). Geographically, the highest provincial prevalence was observed in Taiwan (66.5%), while the lowest was identified in Henan (6.6%). At the regional level, Eastern China had the highest prevalence (40.3%), close to the national average in Northwestern China (31.2%), and the lowest in Central China (6.6%).

**Conclusions:**

The pooled prevalence of myopia among Chinese children and adolescents was 30.1%, with a pronounced increase by educational stage. A slightly higher prevalence was observed in females. Geographically, Eastern China had the highest burden, while Central China had the lowest. These findings highlight marked age, sex, and regional disparities, providing evidence for targeted public health interventions.

**Registration:**

PROSPERO: CRD420251236626.

Myopia, or nearsightedness, is a common refractive error that is becoming a significant public health challenge worldwide. If not effectively treated on time, it can progress to high myopia [1,2], considerably increasing individuals’ risk of developing complications such as cataract, retinopathy, myopic macular degeneration, and glaucoma. These conditions may lead to irreversible vision impairment, placing a substantial long-term burden on individuals, families, and society [3,4]. Myopia has been particularly prevalent in East Asia, with Chinese children and adolescents carrying a substantial portion of this burden [5,6].

While several population- or school-based studies, and even some meta-analyses, have attempted to estimate the prevalence of myopia among Chinese children and adolescents, the comparability of their findings has been limited by inconsistent diagnostic criteria. For example, many investigations have relied on non-cycloplegic refraction, which may overestimate the true prevalence due to unresolved accommodation tension [7,8]. Cycloplegic refraction is considered the ‘gold standard’ for diagnosing myopia in this population, as it effectively paralyses the ciliary muscle to obtain a static refractive measurement that reflects the true refractive error, thereby ensuring the accuracy and comparability of epidemiological data [9,10]. However, few studies have utilised this ‘gold standard’, and even fewer have performed geographically specific analyses. In fact, researchers have not yet examined how the prevalence of myopia differs across China’s provinces and regions or how it is influenced by macro-level factors such as environment, socioeconomic status, and the distribution of educational resources.

To address this gap and generate useful data for developing targeted policies and interventions, we conducted a systematic review and meta-analysis of epidemiological surveys of myopia among Chinese children and adolescents that were based on cycloplegic refraction criteria. Our goal was to estimate the prevalence of myopia in the country, but also to determine whether it varies by sex, age, time periods, province, and major geographical region.

## METHODS

We preregistered the protocol for this study at PROSPERO (CRD420251236626) and reported our findings in accordance with the PRISMA guidelines [11].

We retrieved literature published between 1 January 2020 and 3 March 2025 from PubMed, Web of Science, Science Direct, CNKI, and Wanfang using database-adapted search strategies (Table S1 in the **Online Supplementary Document**). Based on previous literature [7,12], we included population-based or school-based studies that reported, either in English or Chinese, on the prevalence of myopia among Chinese children and adolescents in China, provided they had clearly defined the diagnosis criteria. We excluded studies using non-cycloplegic refraction, those that had a response rate <70%, and those with a sample of <500 participants.

### Data extraction

Two reviewers (HG and JQM) first independently screened the literature in two stages (title/abstract, full text), after which they assessed quality of the included studies and extracted the relevant data in duplicate, resolving any discrepancies through discussion. They then extracted the following data in duplicate into an Excel spreadsheet: first author, publication year, participant size, prevalence of myopia, sex, age, time of myopia assessment, and geographical location (province and region within China). All discrepancies were resolved through discussion.

For subgroup analyses, we categorised age groups into four levels: kindergarten (under 6 years), primary school (6–12 years), junior high school (13–15 years), and high school (16–18 years), based on descriptions in the original studies and official Chinese regulations. we categorised geographical regions within China according to provincial-level administrative divisions and their integrated natural and socioeconomic profiles [13] into seven major areas: North China, Northeast China, East China, Central China, South China, Southwest China, and Northwest China.

### Statistical analysis

We calculated the prevalence of myopia and its corresponding 95% confidence intervals (CIs) using a random-effects model, with weighting performed using the inverse variance method. We assessed heterogeneity using Cochran’s Q and the *τ*^2^ statistic. To further explore potential sources of heterogeneity, we performed meta-regression analyses with time and sex as covariates. We evaluated publication bias using Egger’s and Begg’s test, with a significance level set at *P* < 0.05. When more than 10 studies were included in the meta-analysis, we employed funnel plots to assess publication bias. We performed sensitivity analysis on all included studies. We also performed subgroup analyses based on sex, age, time of myopia assessment, and geographical regions. For the subgroup analysis by examination time, we included only studies reporting data for a single, defined calendar year to ensure precise temporal categorisation; studies reporting data collected over a multi-year period were included in the overall pooled estimate, but excluded from this specific subgroup analysis.

We performed all analyses in *R*, version 4.5.0 (R Core Team, Vienna, Austria) with the ‘meta’ package. A *P*-value <0.05 was set as the threshold for statistical significance.

## RESULTS

**Figure 1:**
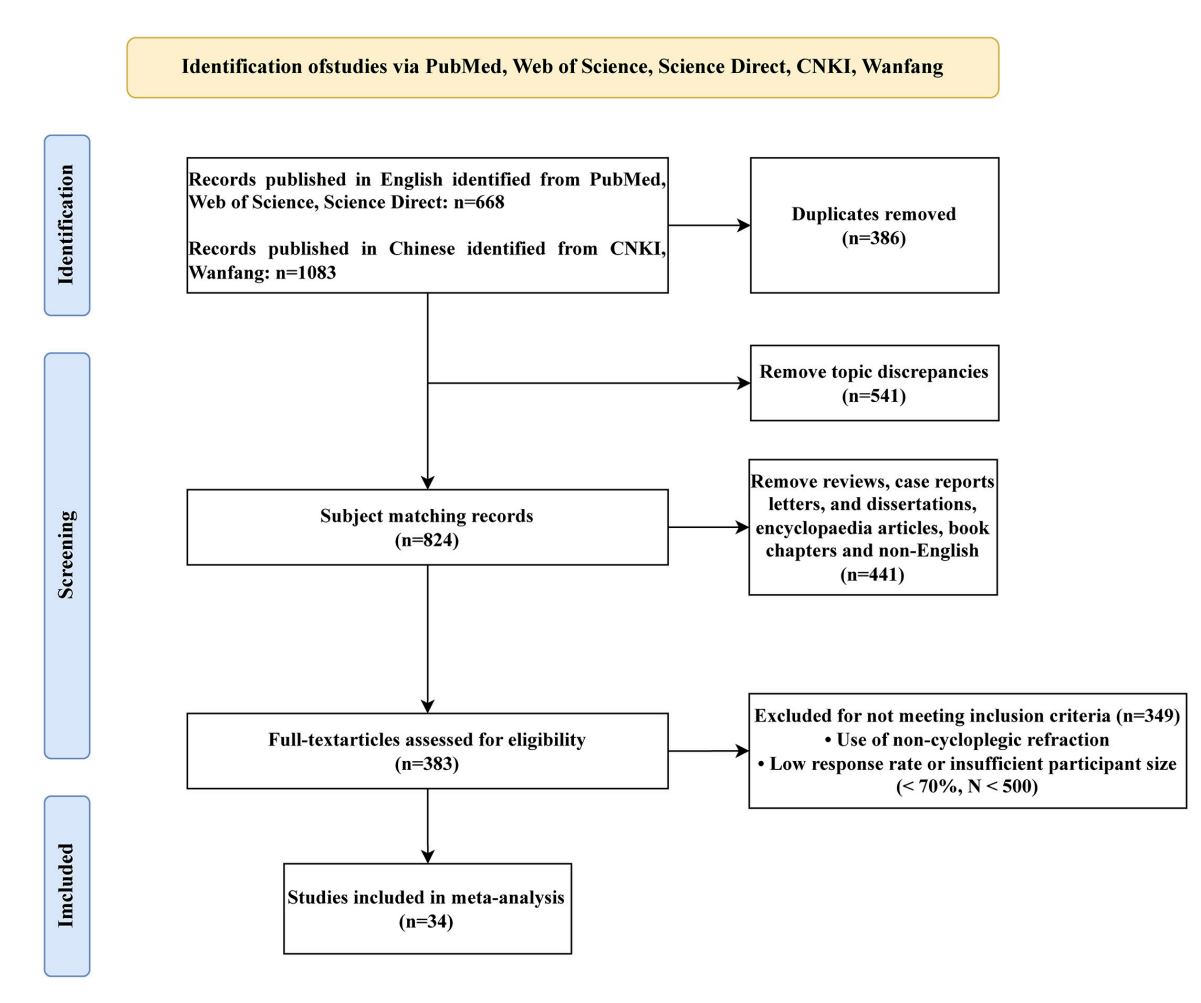
Flowchart of the literature search and collection.

The search retrieved 1751 records, of which 386 were removed after deduplication. The remaining 1365 records underwent a title/abstract screening, where 541 were removed. A further 790 records were excluded (441 non-original research articles and 349 studies that did not meet all criteria) were excluded during full-text screening, leaving 34 articles for analysis (**Figure 1**). These 34 studies collectively encompassed 139 765 children and adolescents with myopia, of whom 123 042 participants contained detailed grade-level information, 75 086 included sex-specific data, 133 106 provided specific visual examination time points, and all 139 765 contained detailed geographic distribution information.

### Myopia prevalence rate

The meta-analysis of 139 765 participants determined the overall prevalence of myopia to be 30.1% (95% CI = 23.4–37.2) (Figure S1 in the **Online Supplementary Document**). The pooled prevalence in the sex-based subgroup analysis of 75 086 participants (Figure S2 in the **Online Supplementary Document**) was 26.0% (95% CI = 16.6–36.7) among males (n = 39 003) and 28.6% (95% CI = 18.0–40.6) among females (n = 36 083). The meta-regression analysis showed no statistically significant association between sex and myopia prevalence (regression coefficient = 0.254, OR = 1.289; *P* = 0.773).

**Figure 2:**
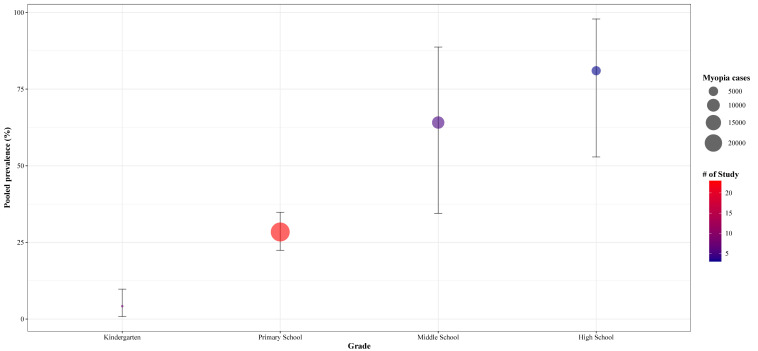
Meta-analysis of the pooled prevalence of myopia by age among Chinese children and adolescents.

The analysis by educational stage (Figure S3 in the **Online Supplementary Document**) encompassed 123 042 participants categorised into four subgroups: kindergarten (n = 13 725), primary school (n = 88 790), junior high school (n = 14 657), and high school (n = 5870). The prevalence of myopia increased markedly with higher educational stages, rising from 4.2% (95% CI = 0.8–9.8) in kindergarten children to 28.4% (95% CI = 22.4–34.8 ) in primary school, 64.1% (95% CI = 34.5–88.7) in junior high school, and 81.0% (95% CI = 52.9–97.9) in high school students (**Figure 2**).

**Figure 3:**
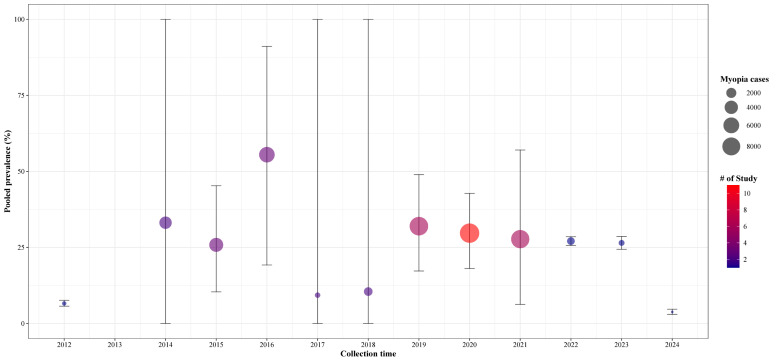
Meta-analysis of the pooled prevalence of myopia among Chinese children and adolescents over time.

The subgroup analysis by time periods, which included 133 106 participants (Figure S4 in the **Online Supplementary Document**), showed that the prevalence of myopia increased gradually from 6.6% in 2012, peaked at 57.5% (95% CI = 19.2–91.1) in 2016, and then declined to 10.5% (95% CI = 0.0–39.1) in 2018. From 2019 onward, the prevalence stabilised, fluctuating around 30% (**Figure 3**). The meta-regression analysis (Figure S5 in the **Online Supplementary Document**) showed no statistically significant linear association between time and myopia prevalence (regression coefficient = −0.012, *P* > 0.443).

The subgroup analysis by province included data from 17 provincial-level administrative regions across China (Figure S6 in the **Online Supplementary Document**). Here, we observed the highest prevalence in Taiwan (66.5%; 95% CI = 23.7–97.1), followed by Yunnan (55.8%; 95% CI = 40.5–70.6), Fujian (48.4%; 95% CI = 46.7–50.0), Anhui (46.0%; 95% CI = 12.2–82.1), Xinjiang (44.9%; 95% CI = 15.2–76.7), Tianjin (38.6%; 95% CI = 34.6–42.6), Shandong (37.5%; 95% CI = 36.0–39.0), Zhejiang (36.9%; 95% CI = 35.3–38.3), Hainan (25.5%; 95% CI = 6.3–51.9), Hong Kong (25.1%; 95% CI = 22.3–27.9), Jiangsu (25.0%; 95% CI = 22.8–27.3), Beijing (23.2%; 95% CI = 7.5–44.3), Guangdong (19.8%; 95% CI = 18.2–21.4), and Qinghai (18.9%; 95% CI = 0–81.69). Henan (6.6%; 95% CI = 5.7–7.5), Xizang (9.3%; 95% CI = 3.9–16.7) and Shanghai (10.5%; 95% CI = 1.8–25.0) had comparatively lower prevalence rates (Figure S7 in the **Online Supplementary Document**). Based on data from six of the seven major geographic regions, we found that East China had the highest prevalence (40.3%; 95% CI = 25.9–55.7), while Central China had the lowest (6.6%; 95% CI = 5.7–7.5). The prevalence in Northwest China (31.2%; 95% CI = 7.6–61.8) was closest to the national estimate of 32.0%. The remaining three regions – North China (24.7%; 95% CI = 7.8–47.0), South China (24.7%; 95% CI = 20.9–28.7), and Southwest China (25.0%; 95% CI = 2.0–61.7) – showed intermediate prevalence rates (Figure S8 in the **Online Supplementary Document**).

### Publication bias and sensitivity analysis

The assessment of publication bias, conducted using Begg’s and Egger’s tests, indicated no significant bias for the pooled prevalence of myopia (*P* > 0.05). We confirmed this by visually inspecting the funnel plot of the funnel plot, which showed an approximately symmetrical distribution of effect estimates (Figure S9 in the **Online Supplementary Document**). We further performed a leave-one-out sensitivity analysis and found that the results did not change significantly from the initial analysis, suggesting a reasonable level of homogeneity across the included studies.

## DISCUSSION

This meta-analysis, based exclusively on cycloplegic refraction data from articles published between 1 January 2020 and 3 March 2025, estimated the prevalence of myopia among children and adolescents in China. It also stratified these estimates across different age groups, genders, geographical regions, provincial areas, and time periods.

Our meta-analysis showed the overall myopia prevalence to be 30.1%, which is consistent with the findings of Pan *et al*. [12], who identified a prevalence of 36.6%. Aligning with previous studies in Asian populations [14,15] and with the study by Rose *et al*. [16], we found a slightly higher myopia prevalence in females compared to males (28.6% *vs*. 26.0%). The higher prevalence among girls may be attributed to various factors, including longer time spent on near-work activities and less time spent outdoors [17], and to differences in social behavioural factors and hormone levels [18]. (36.6%). We also found that the prevalence increase with educational stages, with highest rates in high school (81.0%), followed by junior high (64.1%) and primary school (28.4%) students, and the lowest in kindergarten children (4.2%). These findings highlight the importance of focusing on critical high-risk periods for myopia progression, especially during key transitional grades within the Chinese education system, such as grades 6, 9, and 12 [19]. The marked rise in prevalence observed during school age, particularly prior to adolescence, underscores a critical window for implementing behavioural interventions targeting modifiable risk factors established in young populations, such as insufficient time outdoors and increased digital screen use [20,21]. Time trend analysis indicated that prevalence peaked in 2016 (57.5%), with fluctuating patterns observed in other years. A study based on projections from three models predicted that by 2050, 61.3% and 17.6% of children in China will have myopia and high myopia, respectively [12].

In July 2021, China introduced the ‘Double Reduction’ policy that, in targeting students in grades one through nine, sought to alleviate the burden of in-school assignments and off-campus tutoring, standardise after-school training institutions, limit daily homework duration, and promote outdoor activities. These measures were designed to advance the balanced development of quality education and compulsory schooling, while also contributing to the improvement of students’ visual health. A longitudinal study conducted by Hu *et al*. [22] followed over 130 000 students in two eastern Chinese cities and identified a significant slowdown in myopia progression in their right eyes one year after the implementation of the policy. Specifically, the spherical equivalent refraction improved by 0.058–0.115 D, while the axial length decreased by 0.009–0.163 mm. Furthermore, students with mild to moderate myopia exhibited more pronounced improvements. Taken together, these findings of Hu *et al*. [22] provide clear evidence that reducing academic burdens can effectively delay the progression of myopia, thereby offering empirical support for the role of policy interventions in preventing and controlling myopia among adolescents. Yet despite the implementation of such policies, the burden of myopia continues to present a significant national public health burden [23].

We found substantial geographical variations in the prevalence of myopia, with Taiwan, Fujian, and Yunnan showing one of the highest prevalence rates, and Henan having the lowest This suggests that the prevalence of myopia in China varies by regions, likely due to the influence of environmental, socioeconomic, educational, and behavioural factors. Among the six geographic regions, East China demonstrated consistently high myopia rates, a finding consistent with the report by Wang *et al*. [24]. The elevated prevalence in this economically developed region aligns with studies in Asian contexts, where higher socioeconomic status and associated educational pressures are frequently linked to a greater risk of myopia [25]. Multiple studies have indicated a positive correlation between academic pressure and the related visual fatigue and the incidence of myopia [26–30]. Students in East China face considerable academic demands, engaging in prolonged near-work activities such as extensive homework and extracurricular tutoring, while high degree of urbanisation in the region and widespread use of electronic devices have significantly increased screen time, likely further contributing again to the high prevalence of myopia [31,32]. Additionally, extensive engagement in indoor activities coupled with insufficient outdoor time (the latter being a well-established risk factor for myopia) may further contribute to the risk in such environments [33,34].

The myopia prevalence in Northwest China was close to the national average. With a moderate level of economic development and educational resource allocation, this region experiences relatively less academic competition pressure compared to more developed areas [35]. Consequently, students’ have a lower visual load compared to other regions, which may help mitigate the onset and progression of myopia [36,37]. However, with ongoing economic growth and lifestyle changes, Northwest China also faces myopia prevention and control challenges, such as increasing use of electronic devices and reduced outdoor activity time due to urbanisation [38]. Central China, meanwhile, had the lowest prevalence of myopia. The region is characterised by a distinct four-season climate, featuring a relatively short winter; this facilitates ample exposure to sunlight and offers more opportunities for outdoor activities, both of which are known to mitigate the risk of myopia onset and progression [33,39–42].

This high prevalence of myopia and its distinct regional distribution patterns among Chinese children and adolescents should be considered within a broader global context. China’s burden of myopia aligns with trends observed in other East Asian societies such as Singapore and South Korea, underscoring the effects of high-intensity educational models and highly urbanised environments [32,43]. In contrast, regions like Northern Europe and Australia, which emphasise outdoor activity cultures and maintain relatively less pressurised educational systems, report significantly lower prevalence rates compared to China [44,45]. These findings suggest that public health policies and myopia intervention measures should be tailored to local conditions. In regions with a high prevalence of myopia, such as East China, visual health management should be strengthened by reducing students’ academic burden, rationally controlling time spent on near-work activities, and increasing outdoor activity time to effectively lower the incidence of myopia. Efforts in other regions should also be made to actively promote lifestyles and behavioural habits tailored to local contexts.

Besides such public health interventions, considerable progress has been made with direct optical and pharmaceutical treatment. Specifically, spectacle lenses designed to manage peripheral defocus and orthokeratology have been demonstrated to effectively delay axial elongation and myopia progression by modifying relative peripheral hyperopic defocus [46,47], while low-concentration atropine eye drops (such as 0.01%) have emerged as an important option for managing rapidly progressive myopia [48,49]. Future research should explore the synergistic effects of combining optical and pharmaceutical therapies, which could represent a key direction for enhancing intervention efficacy. Policy measures should extend beyond reducing academic burdens to actively fostering ‘vision-friendly’ environments, such as by integrating optimised classroom lighting and expanded outdoor activity spaces into school construction standards, or cultivating community-wide initiatives that encourage outdoor time.

A key strength of this study is its strict limitation to cycloplegic refraction data, which minimizes the potential overestimation of myopia prevalence common in non-cycloplegic settings and provides more reliable baseline estimates. However, several limitations should be acknowledged. First, the age range of the participants in the included studies was relatively broad, with considerable heterogeneity among studies. This variability may introduce bias into the pooled estimates of myopia prevalence in the meta-analysis, particularly affecting the accurate assessment of myopia development trajectories during critical growth periods (such as the transition from primary to junior high school) and thus leading to biased prevalence estimates in certain age subgroups. Second, our analysis only included 34 articles, with some provinces or time periods being underrepresented. This may have resulted in unstable prevalence estimates in regions with extreme values, such as Taiwan and Henan. Furthermore, most of the included studies did not consistently or sufficiently measure and report on environmental and behavioural factors, such as daily outdoor activity time, duration of near-work activities, and light exposure, limiting us from determining how these factors impacted our findings. Future research should adopt high-quality, large-participant, multi-centre prospective cohort designs. A specific focus of both future cohort studies and tailored interventional research should be the early onset of myopia in younger children. There is also a need to promote standardised diagnostic procedures and environmental exposure assessments, and to conduct in-depth interventional studies tailored to different regions, age groups, and ethnic populations. Despite these limitations, our study provides valuable epidemiological evidence on the current prevalence of myopia and in children and adolescents in China. It underscores the need for multi-level, precise interventions and policies that account for geographical and regional differences, and individuals socioeconomic characteristics and behaviours.

## CONCLUSIONS

This meta-analysis, based on epidemiological studies utilised cycloplegic refraction to diagnose myopia among children and adolescents in China, estimated the national prevalence of the condition to be 30.1%. These rates increased significantly with educational stages, exceeding 80% in high school students. We further found substantial geographical differences, with the highest prevalence in East China (40.3%) and the lowest in Central China (6.6%) We also found a slightly higher prevalence in females than in males and identified fluctuations in temporal trends. These findings provide key data for evaluating the effectiveness of current myopia prevention efforts and formulating more targeted and region-specific public health strategies in the future.

## Additional material


Online Supplementary Document
