# Supplementary material for: Prevalence of myopia in Chinese children and adolescents: a systematic review and meta-analysis
Source: J Glob Health. 2026 Mar 20;16:04056. doi: 10.7189/jogh.16.04056 (PMC13002174; doi:10.7189/jogh.16.04056)
Supplement: Online Supplementary Document [file jogh-16-04056-s001.pdf]

Supplement to: Gao H, Ma J, Liu Z, Wang J, Wang W, Ye L. Prevalence of myopia in Chinese children and adolescents: a systematic review and meta-analysis. J Glob Health. 2026;16:04056.

Table S1. Systematic search strategy for identifying studies on myopia prevalence

Database	URL	Search strains	Number of records
PubMed	https://pubmed.ncbi.nlm.nih.gov/	(“Myopia”[MeSH] OR myopia[Title/Abstract]) AND (“Prevalence”[MeSH] OR prevalence[Title/Abstract]) AND (“China”[MeSH] OR “Hong Kong”[MeSH] OR “Taiwan”[MeSH] OR “Macau”[MeSH] OR China[Title/Abstract] OR “Hong Kong”[Title/Abstract] OR Taiwan[Title/Abstract] OR Macau[Title/Abstract])	115
Science Direct	https://www.sciencedirect.com/	Title/abstract/key words = (myopia AND prevalence AND (China OR “Hong Kong” OR Macau OR Taiwan))	18
Web of Science	https://www.webofscience.com/wos/alldb/basic-search	TS = (myopia AND prevalence AND (China OR “Hong Kong” OR Macau OR Taiwan))	535
China National Knowledge Infrastructure	https://chn.oversea.cnki.net/index/	Subject = (myopia AND prevalence AND (China OR “Hong Kong” OR Macau OR Taiwan))[a]	372
Wanfang	https://wanfangdata.com.cn/index.html	Subject = (myopia AND prevalence AND	711

	(China OR "Hong Kong" OR Macau OR Taiwan)[a]	

*Corresponding Chinese words were used.

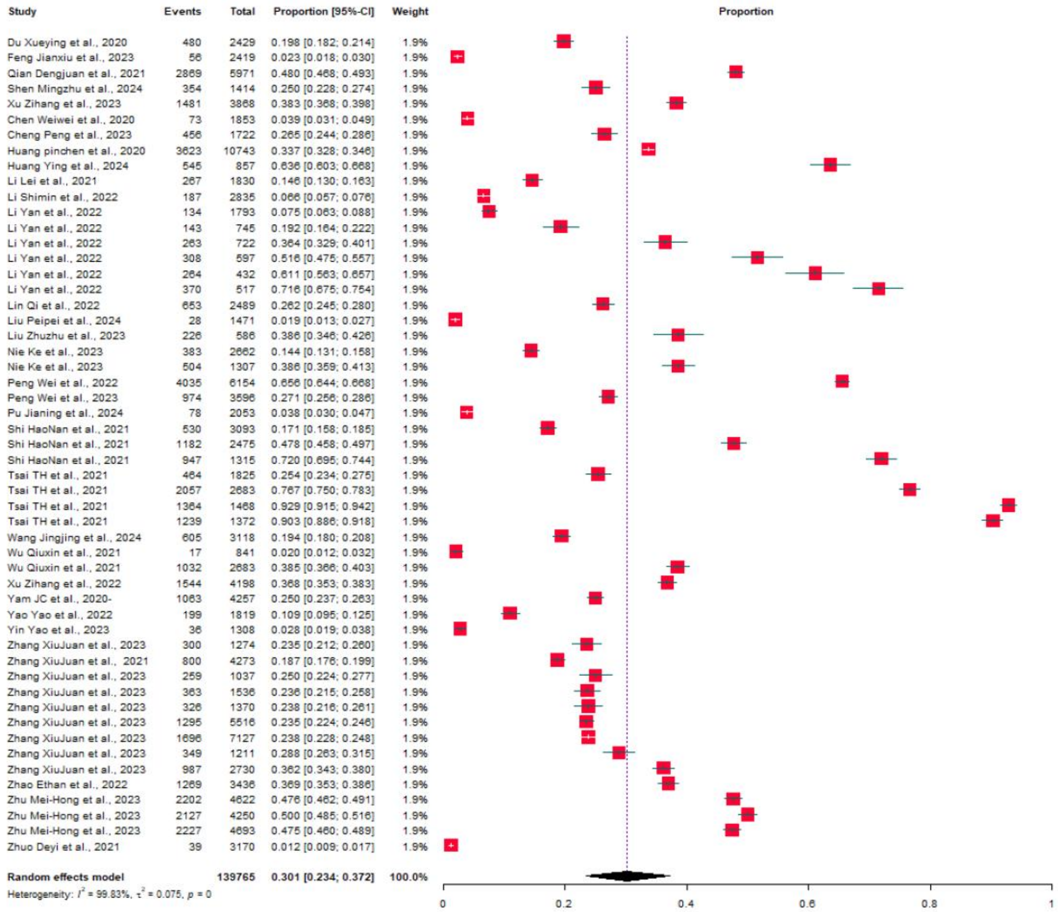

Supplementary Figure S1. Meta-analysis of myopia prevalence among children and adolescents in China

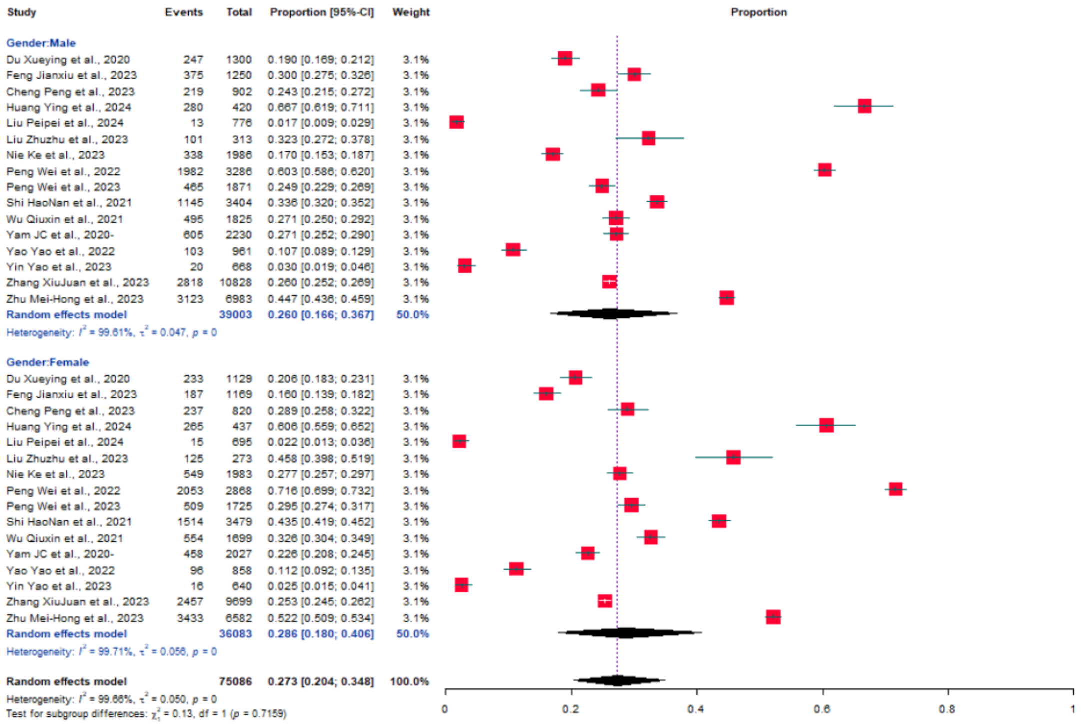

Supplementary Figure S2. Meta-analysis of myopia prevalence in children and adolescents of different sexes in China

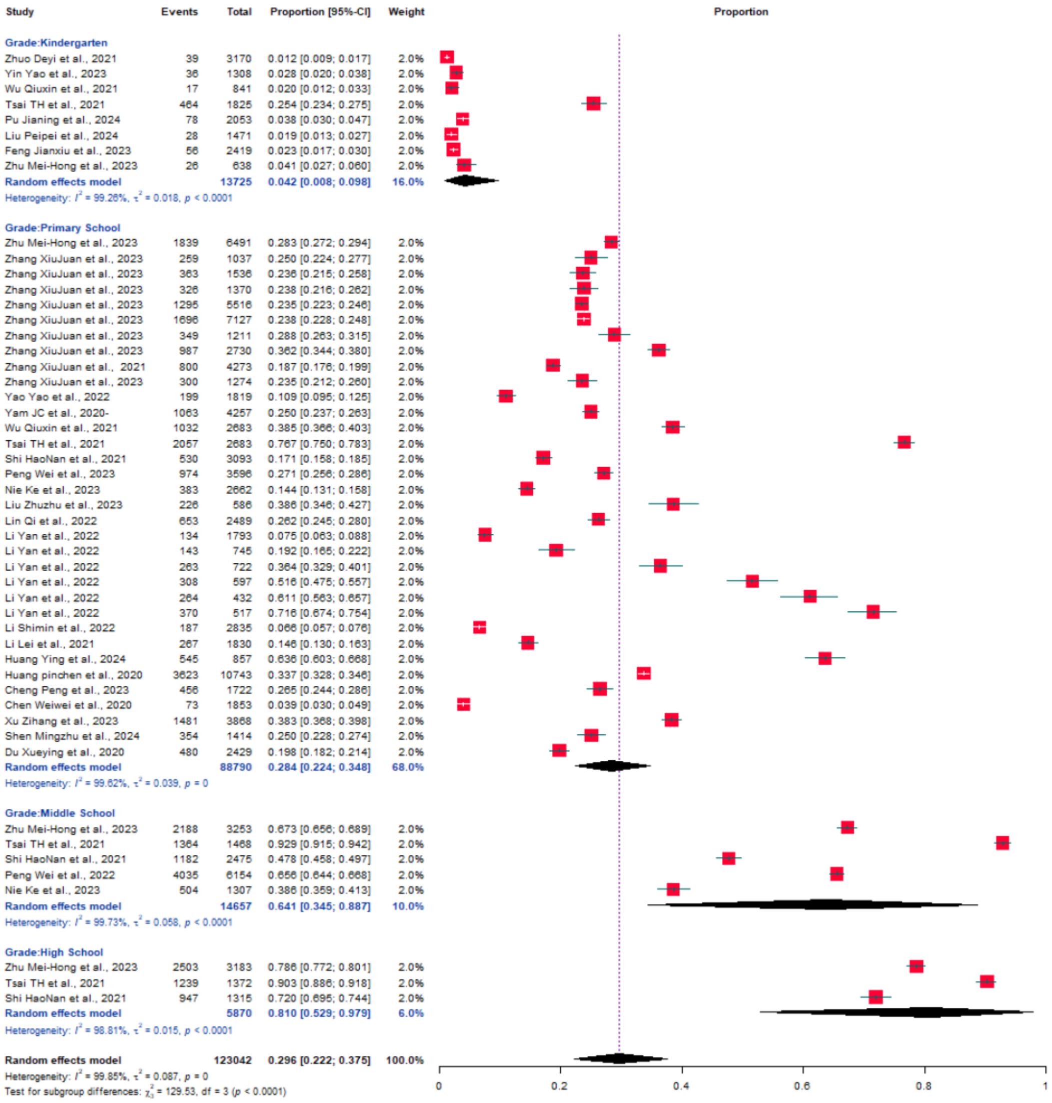

Supplementary Figure S3. Meta-analysis of myopia prevalence in children and adolescents of different ages in China

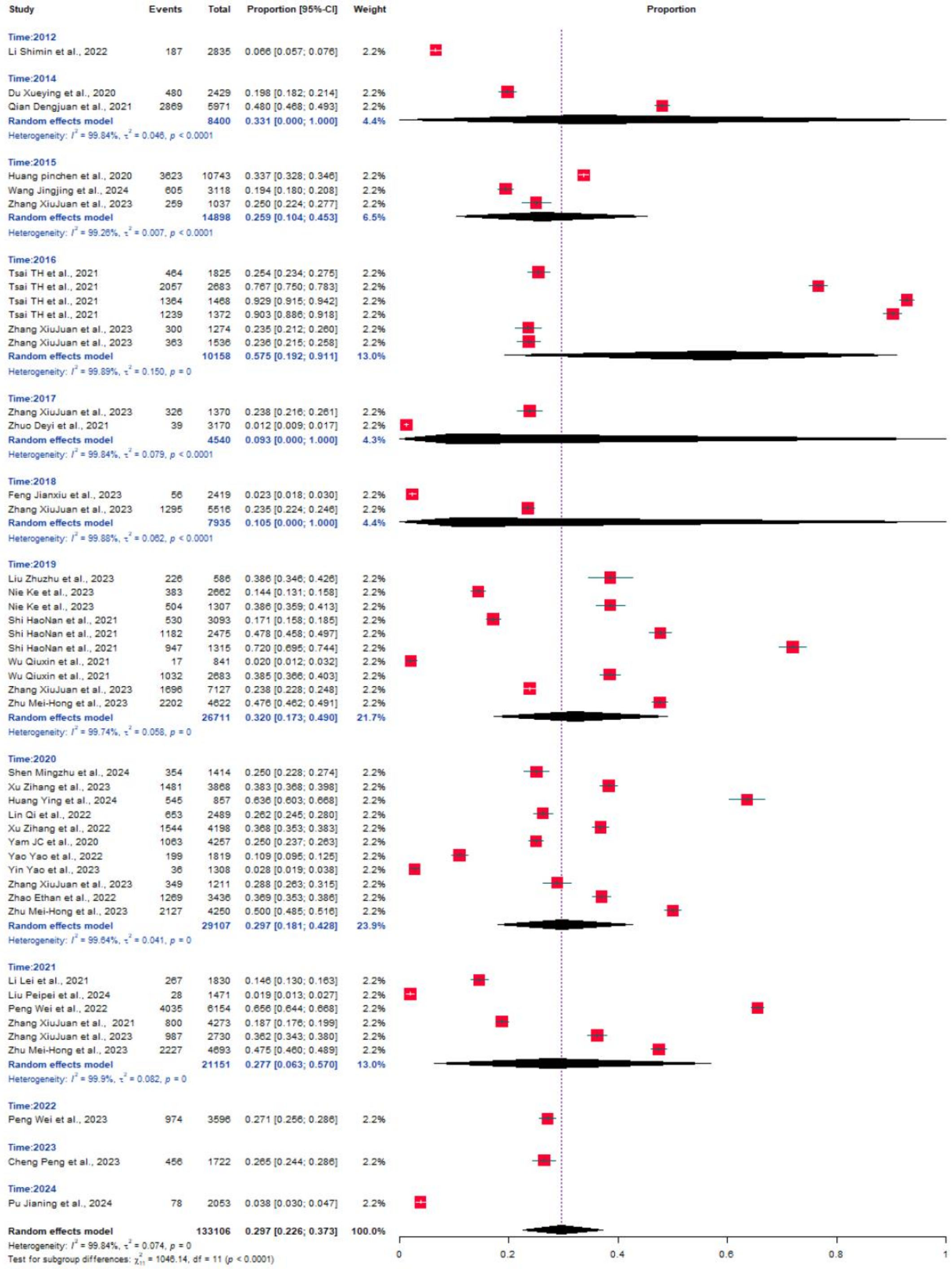

Supplementary Figure S4. Meta-analysis of myopia prevalence in children and adolescents at different time points in China

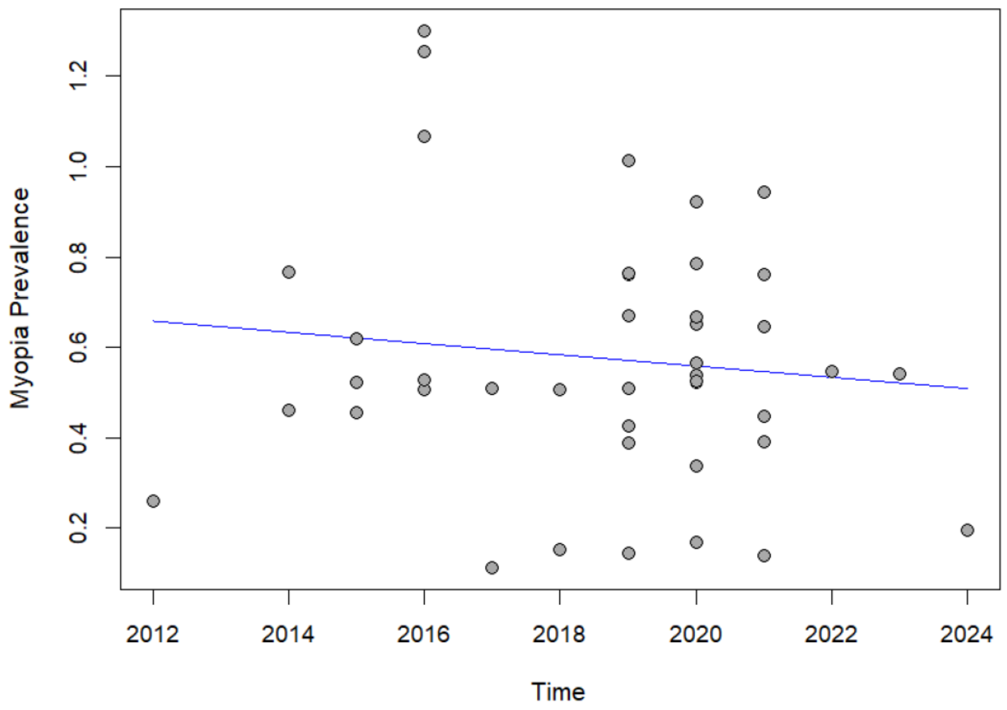

Supplementary Figure S5. Meta-regression of myopia prevalence over time

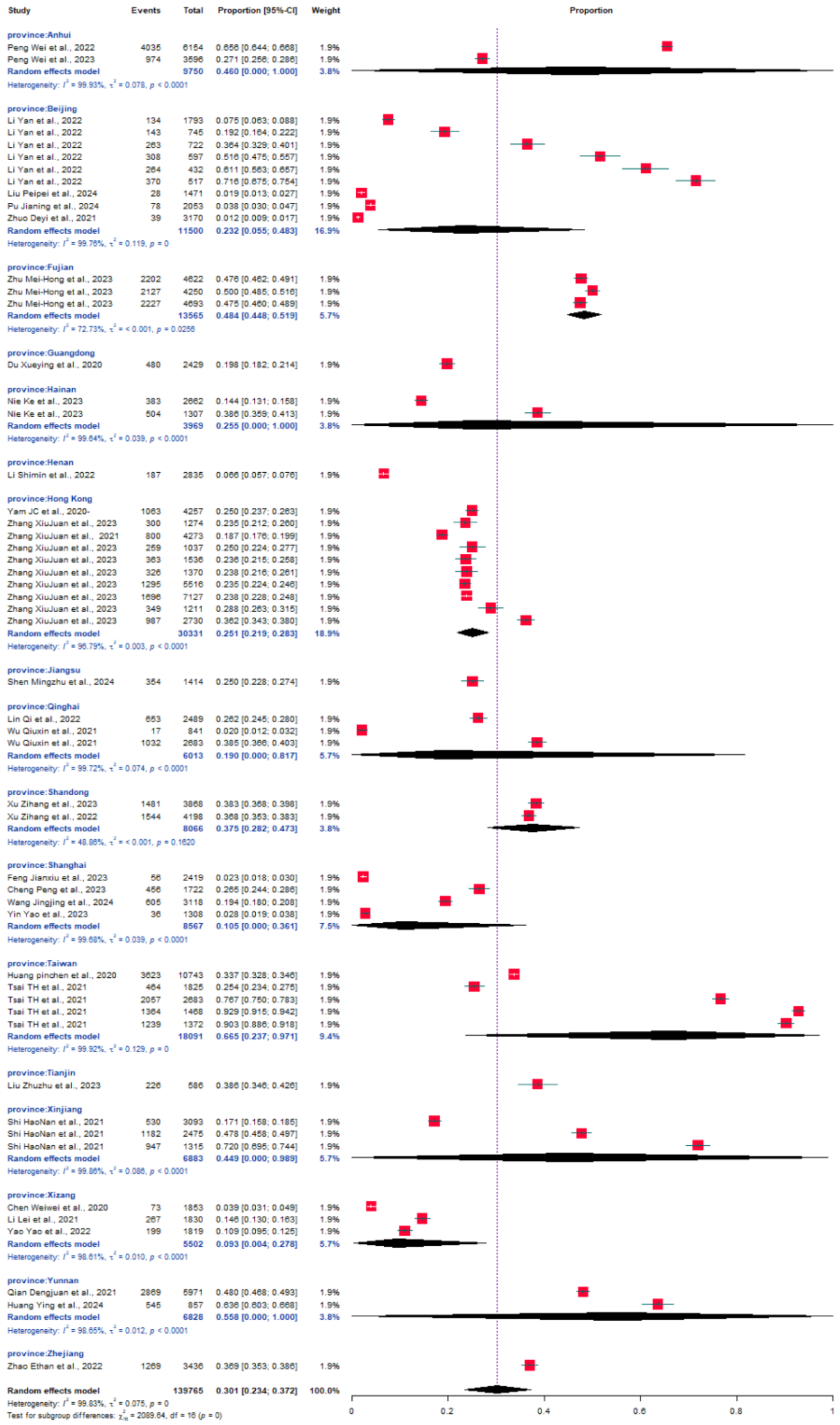

Supplementary Figure S6. Meta-analysis of the pooled prevalence of myopia by province among Chinese children and adolescents.

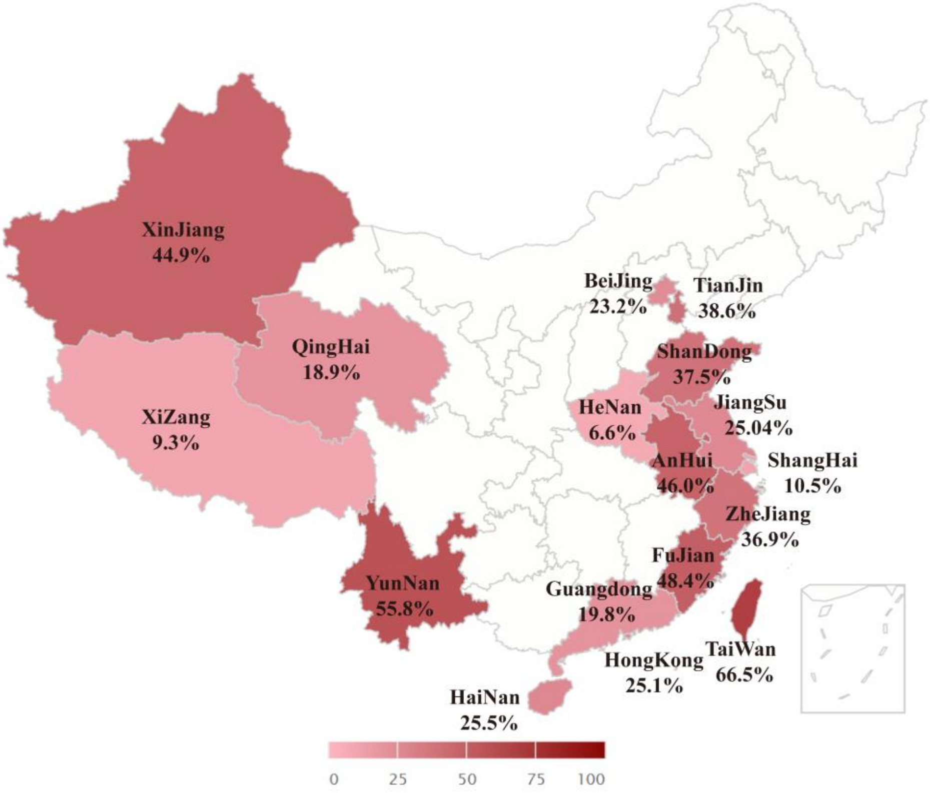

Supplementary Figure S7. Meta-analysis of the pooled prevalence of myopia by province among Chinese children and adolescents

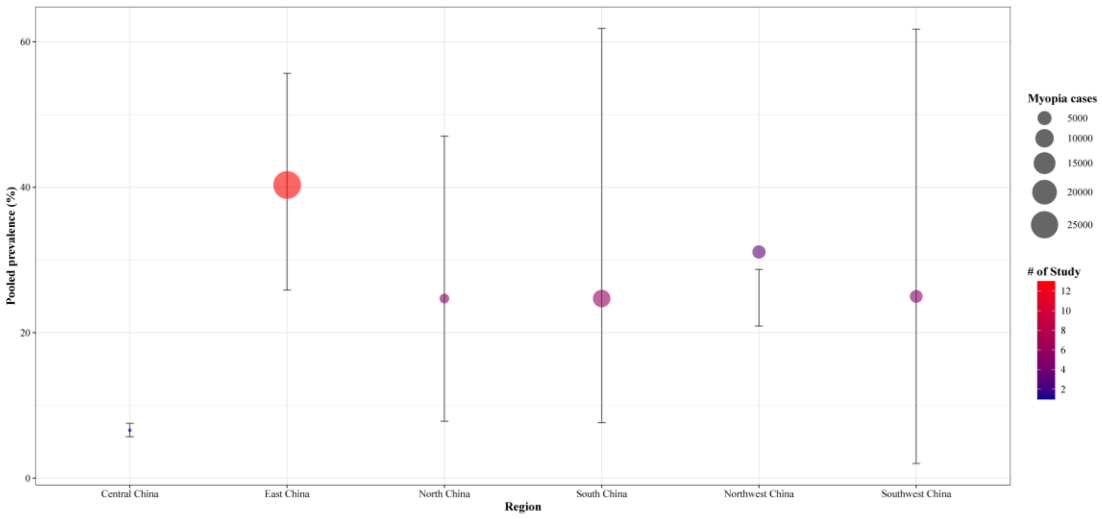

Supplementary Figure S8. Meta-analysis of the pooled prevalence of myopia by region among Chinese children and adolescents.

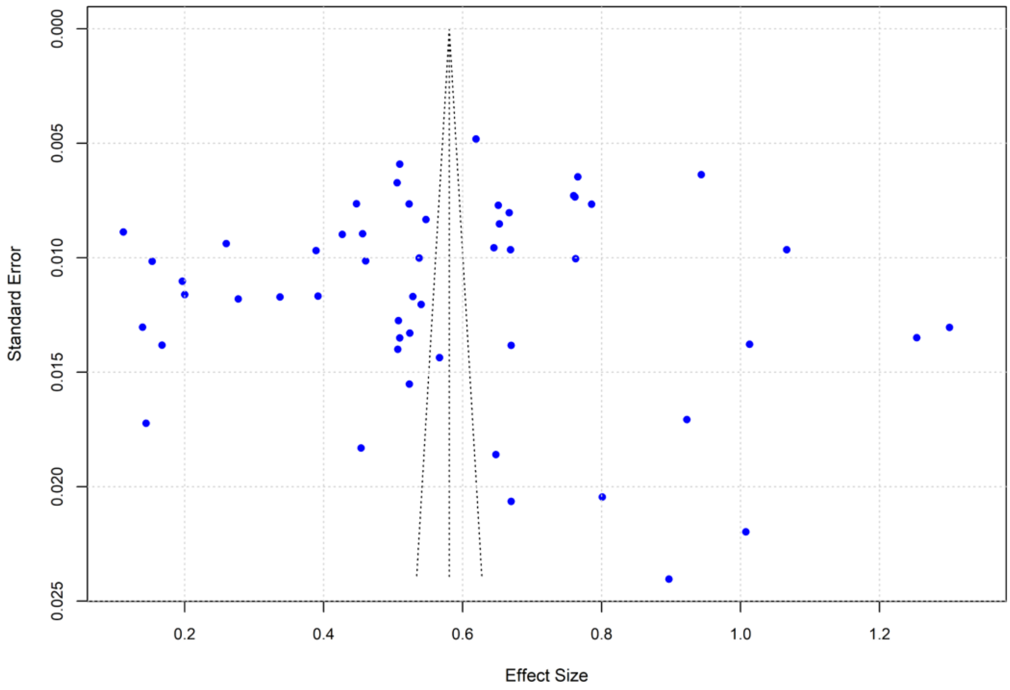

Supplementary Figure S9. Funnel plot to assess publication bias in the meta-analysis of myopia prevalence
